# Encephalitis in Thailand: A Neglected Disease Increasingly Caused by Enterovirus

**DOI:** 10.3390/tropicalmed6030117

**Published:** 2021-06-30

**Authors:** Pasin Hemachudha, Sininat Petcharat, Soawapak Hinjoy, Abhinbhen W. Saraya, Thiravat Hemachudha

**Affiliations:** 1 Thai Red Cross Emerging Infectious Diseases Health Science Centre, World Health Organization Collaborating Centre for Research and Training on Viral Zoonoses, King Chulalongkorn Memorial Hospital, Faculty of Medicine, Chulalongkorn University, Bangkok 10330, Thailand; cakeinarak@hotmail.com (S.P.); abhinbhen@gmail.com (A.W.S.); fmedthm@gmail.com (T.H.); 2 Office of International Cooperation, Department of Disease Control, Ministry of Public Health, Nonthaburi 11000, Thailand; soawapak@gmail.com

**Keywords:** encephalitis, enterovirus, neurological infection, Thailand

## Abstract

From 2013 to 2018, the Thai Red Cross Emerging Infectious Disease–Health Science Center (TRC-EID-HS), in collaboration with the Department of Disease Control (DDC) and the Ministry of Public Health (MOPH) Thailand, conducted encephalitis surveillance. A total of 1700 cerebrospinal fluid (CSF) samples from patients with encephalitis were tested by a predesigned multiplex PCR. Diagnosis was made in 318 cases (18.7%), 86 (27%) of which were caused by Epstein–Barr virus (EBV), 55 (17.3%) by enteroviruses (EV), 36 (11.3%) by varicella–zoster virus (VZV), 31 (9.7%) by cytomegalovirus (CMV), 25 (7.8%) by herpes simplex virus type 1 (HSV-1), and 20 (6.3%) by human herpesvirus 6 (HHV-6). Results were compared with 3099 CSF samples from patients with encephalitis collected between 2002 to 2012, which were tested by specific PCR assays. Diagnosis was made in 337 (10.9%) of these cases, and 91 (27%) were CMV, 79 (23.4%) were VZV, 72 (21.4%) were EBV, 39 (11.6%) were EVs, 39 (11.6%) were HSV-1, 33 (9.8%) were HSV-2, and 2 (0.6%) were Dengue virus (DENV). The change in the pattern toward EVs as a major cause of viral encephalitis was unexpected, and a change in viral neurotropism may be responsible.

## 1. Introduction

The epidemiology of encephalitis is constantly evolving and posing significant challenges in both diagnosis and management [1]. A well-known cause of acute viral encephalitis includes herpes simplex virus (HSV), varicella–zoster virus (VZV), cytomegalovirus (CMV), and enterovirus (EV) [2,3]. Other types of viral encephalitis found in the tropics are much less common overall but notably include Japanese encephalitis, Rabies, Nipah, West Nile, and Chikungunya [4,5]. The presentation may range from mild flu-like illness, fever, and headache to altered mental status or seizure from widespread parenchymal involvement. Focal neurological deficit commonly occurs, and meningeal irritation is not uncommon. Those who survived are often left with significant morbidity [6,7].

Enterovirus genus encompasses positive-sense, single-stranded RNA virus belonging to the Picornaviridae family. It includes five groups, i.e., coxsackievirus, echovirus, enterovirus, rhinovirus, and poliovirus, based on pathogenesis and serotypes. In turn, the group of enteroviruses includes 15 species, i.e., Enterovirus A to L (EV) and Rhinovirus A to C [8]. EV infections commonly affect children and are usually transmitted via fecal–oral route [3,9]. It can manifest in various ways, from nonspecific flu-like illness and rash to devastating illness such as myocarditis and central nervous system (CNS) infection [2,6,7]. Multiple serotypes are known to possess neurotropism and are capable of penetrating the CNS [10,11]. Enterovirus is also known for its high mutation rate from an error-prone RNA recombination process [12,13].

Thai Red Cross Emerging Infectious Disease–Health Science Center (TRC-EID-HS) has been working with the Department of Disease Control (DDC) and the Ministry of Public Health (MOPH) on encephalitis surveillance and diagnosis since 2013. The paper present pathogens found in CSF specimen taken from patients with neurological symptoms consistent with encephalitis during 2013–2018 and focusing on enterovirus genus. We then compare results to those collected from 2002 to 2012.

## 2. Materials and Methods

### 2.1. Patients

**Figure A1:**
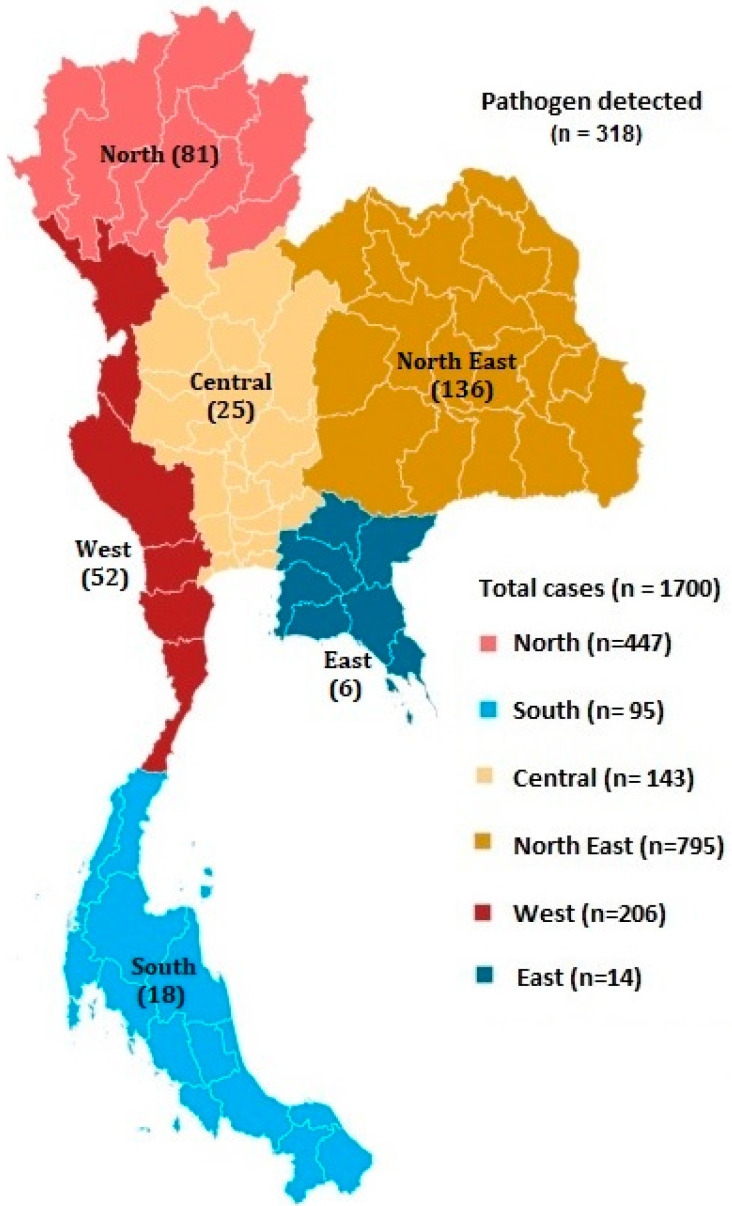
Geographic distribution of patients with suspected encephalitis in Thailand from 2013 to 2018. A map showing number of positive cases in different regions.

TRC-EID-HS has been providing molecular service including virological screening with real-time polymerase chain reaction (RT-PCR) since 2002. CSF samples were sent to us from 30 provinces (40 hospitals) in Thailand from patients with suspected encephalitis (see Figure A1). Collaboration with DDC started in 2013, and we received 1700 CSF samples between 2013 to 2018 for the meningoencephalitis RT-PCR panel.

Viral encephalitis was defined as fever, headache, altered mental status, decreased level of consciousness, or seizures with or without focal neurological deficit. All enrolled patients presented symptoms consistent with encephalitis, regardless of their age, at the discretion of the treating physician.

All samples tested included pan-enterovirus genus (Coxsackie A and B, echoviruses, polioviruses, and EV 68–71, 74–78, and 93–94) and were sequenced for positive HSV-1/2, VZV, CMV, Epstein–Barr virus (EBV), Human herpesvirus 6 (HHV-6), Human parechovirus, *Escherichia coli* K1, *Haemophilus influenzae*, *Listeria monocytogenes*, *Neisseria meningitidis*, *Streptococcus agalactiae*, *Streptococcus pneumoniae*, and *Cryptococcus neoformans/gatti*.

### 2.2. The BioFire FilmArray Meningitis/Encephalitis Panel

The FilmArray meningitis/encephalitis (ME) panel (BioFire Diagnostics, LLC, Salt Lake City, UT, USA) reagent pouch stored all the necessary reagents for sample preparation, reverse transcription PCR, and detection in a freeze-dried format. Prior to a run, the user injected hydration solution and sample combined with sample buffer into the pouch. The FilmArray instrument (bioMérieux, Salt Lake City, UT, USA) carried out the rest. First, the FilmArray extracted and purified all nucleic acids from the sample. Next, the FilmArray performed a nested multiplex PCR. During the first-stage PCR, the FilmArray performed a single, large volume, massively multiplexed reaction. Last, individual single-plex, second-stage PCR reactions detected the products from the first stage PCR. Using endpoint melting curve data, the FilmArray software (CSF v3.0) automatically generated a result for each target in a single report. Tests were conducted on cerebrospinal fluid (CSF) for a variety of pathogens—bacteria: *Escherichia coli* K1, *Haemophilus influenzae, Listeria monocytogenes, Neisseria meningitidis, Streptococcus agalactiae*, and *Streptococcus pneumoniae*; viruses: Enterovirus, HSV-1, HSV-2, VZV, CMV, HHV-6, and Human parechovirus; yeast: *Cryptococcus neoformans/gattii*.

### 2.3. Nucleic Acid Extraction

Nucleic acid was extracted by a manual nucleic acid extractor, the *Nuclisens mini MAG* (bioMérieux, Akzo Nobel, The Netherlands). Then, 0.1–1 mL of CSF specimen was added to 2 mL of lysis buffer to mix and incubated for 10 min at room temperature. Next step, 50 µL of magnetic silica was added for nucleic acid release (worksheet *Nuclisens Magnetic* Extraction, (bioMérieux, Akzo Nobel, The Netherlands); nucleic acid isolation with eluted 50 µL of elution buffer was then stored at −70 °C.

### 2.4. Real-Time Reverse Transcription Polymerase Chain Reaction

Real-time RT-PCR in the amplified product was detected by fluorescent dyes. The Enterovirus R-GENE kit was used to determine Enterovirus A: Coxsackievirus A4, A6 to A8, A10, A14, A16, A16V, and Enterovirus 71, 76; Enterovirus B: Coxsackievirus A9, B1 to B6, and Echovirus 1 to 7, 9, 11 to 21, 24 to 27, 29 to 33, and Enterovirus 69, 74, 75, 77, 78, 93; Enterovirus C: Coxsackievirus A11, A13, A17, A20, A21, A24, A24V, and Poliovirus 1, 2, 3; Enterovirus D: Enterovirus 68, 70, 94. Then, 15 µL of the master mix was automatically pipetted into each glass capillary, adding 10 µL of the nucleic acid template. For the programming of real-time RT-PCR, a LightCycler 2.0 instrument was used according to the following five steps: the first step was a reverse transcription, 50 °C for 5 min per cycle; the second was Taq polymerase activation, 95 °C for 15 min per cycle; the third was amplification with 45 cycles, 95 °C for 10 s, 60 °C for 40 s, wavelength for signal reading, and 72 °C for 25 s. A signal was detected using fluorometer channel 530.

### 2.5. Enteroviruses Family Polymerase Chain Reaction

The serotype of all EV-positive samples was confirmed by identifying 5′-untranslated region (5′-UTR) sequences. Conventional RT-PCR targeting the 5′-UTR gene performed with the primer set 5′ UTR Long-F: GGT CAA GCA CTT CTG TTT CCC and 5’UTR L541-R: GAA ACA CGG WCA CCC AAA GTA STC G for the PCR product of approximately 400 bp. This protocol was developed by PREDICT USAID (Unpublished, Designed at CII). Briefly, 5 µL of total nucleic acid was amplified in a 50 µL reaction using a one-step RT-PCR kit (Qiagen) with the following conditions: 50 °C reverse transcription for 30 min, 95 °C 15 min, then 16 cycles of 94 °C for 45 s, 65 °C for 45 s (−1 °C/cycle), and 72 °C for 45 s. Then, the test was performed in 32 cycles of 94 °C for 45 s, 48 °C for 45 s, and 72 °C for 45 s, and finished with 72 °C for 5 min. Amplicons from the EV-positive band were agarose gel-purified and sequenced. The nucleotide sequence was analyzed using the Basic Local Alignment Search Tool (BLAST).

### 2.6. Ethics Statement

The study was approved by the Ethics Committee, the Institutional Review Board of the Faculty of Medicine, Chulalongkorn University, Bangkok, Thailand (Reference No. 015/2011).

## 3. Results

**Figure 1:**
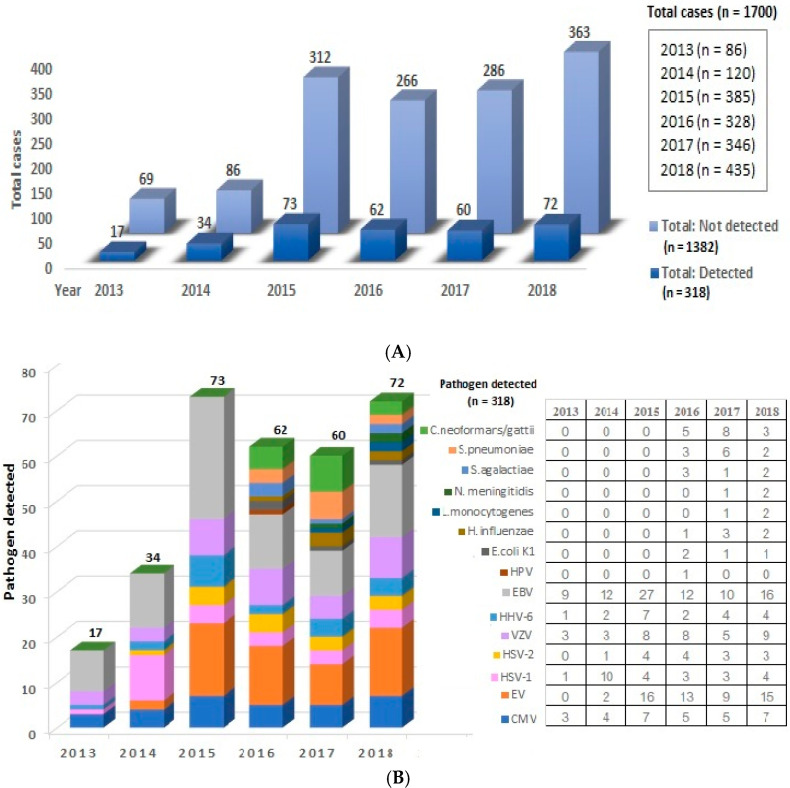
Cause of encephalitis from 2013 to 2018: (**A**) the number of patients with and without pathogen detected by year; (**B**) summary of CSF finding by year.

**Figure 2:**
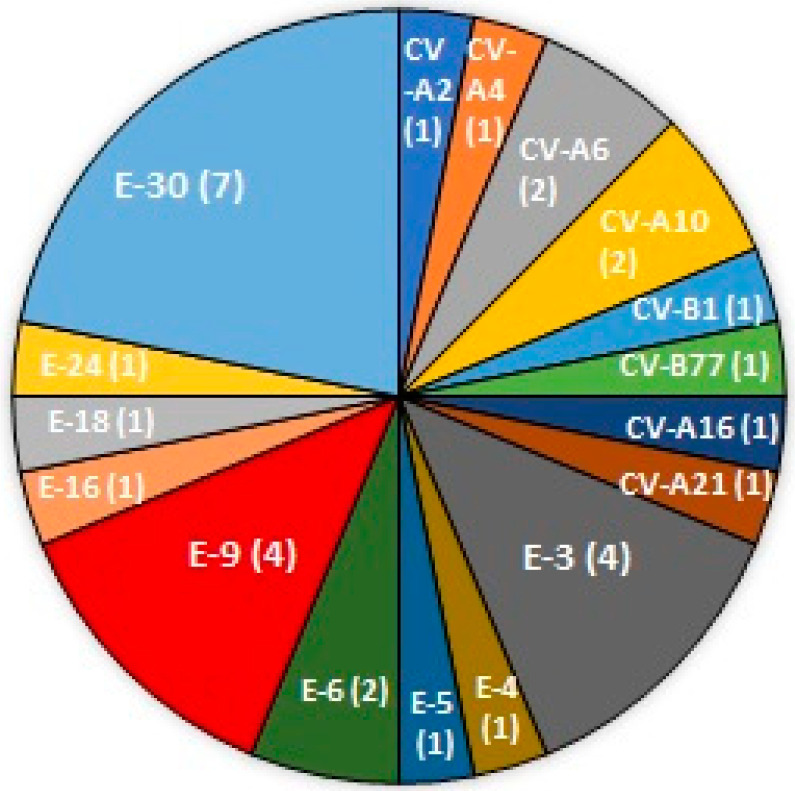
Found serotypes in 32 patients with EV encephalitis. The number of samples is denoted by the number in the pie chart.

A total of 1700 CSF samples received from 2013 to 2018 were retrospectively analyzed, and 318 samples were found positive for pathogens (18.7%), (Figure 1A). The most frequent diagnosis was EBV with 86 samples (27%), followed by enterovirus with 55 samples (17.3%), VZV with 36 samples (11.3%), CMV with 31 samples (9.7%). HSV-1 with 25 samples (7.8%), HHV-6 with 20 samples (6.3%), *Cryptococcus neoformans/gattii* with 16 samples (5%), HSV-2 with 15 samples (4.7%), *Streptococcus pneumoniae* with 11 samples (3.5%), *Haemophilus influenzae* and *Streptococcus agalactiae* equally with 6 samples (1.9 %), *E. coli* K1 with 4 samples (1.3%), *Neisseria meningitidis* and *Listeria monocytogenes* equally with 3 samples (1%), and Human parechovirus with 1 sample (0.3%), (Figure 1B). Only 32 CSF samples positive for EV were adequate for further serotyping, and a total of 17 different enterovirus serotypes were detected, namely, human enterovirus A serotypes Coxsackievirus (CV)-A2, CV-A4, CV-A6, CV-A10, CV-A16, CV-A21, and human enterovirus B serotypes CV-B1, Enterovirus (EV)-B77, Echovirus (E)-3, E-4, E-5, E-6, E-9, E-16, E-18, E-24, E-30 (Figure 2). The most common virus causing EV encephalitis in our study was E-30 with seven samples, followed by E-3 and E-9, both with four samples.

**Figure 3:**
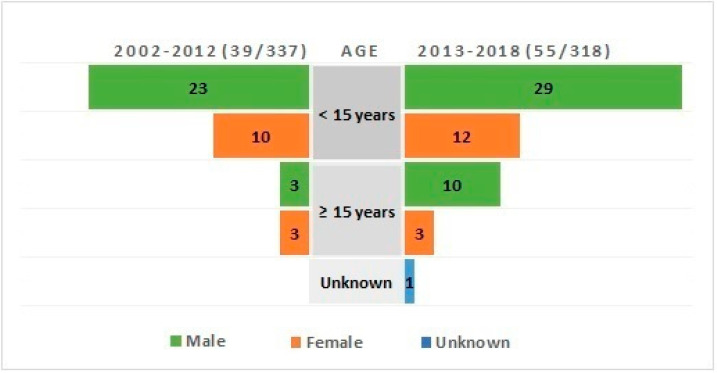
Demographic of individuals with confirmed enterovirus encephalitis in 2002–2012, and in 2013–2018.

Demographics of individuals with EV encephalitis were obtained from the encephalitis RT-PCR request form. Overall, 54 of 55 request forms were filled with the patient’s age, and 13 (24.1%) were adults (Figure 3). As for presenting illness, 37 from 55 request forms were filled with the patient’s symptoms. Of 37 individuals, 26 (70.3%) presented with high temperature, followed by 15 (40.5%) with headache, 12 (32.4%) with seizures and/or spasms, and 7 (18.9%) with altered mental status.

**Figure 4:**
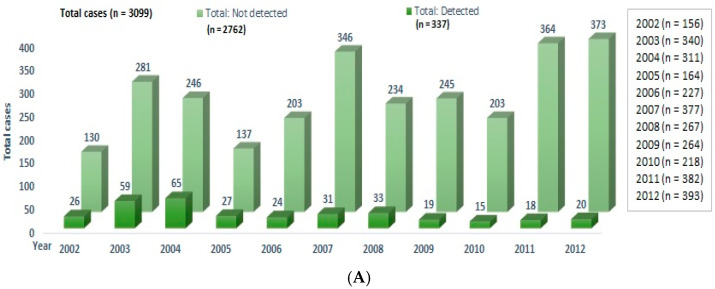

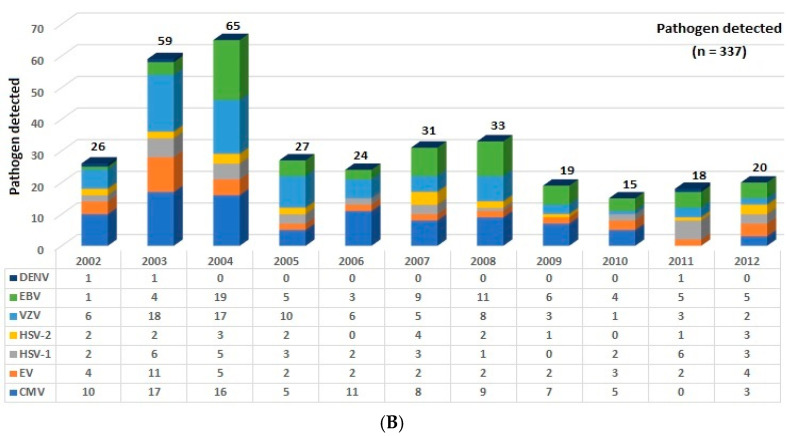
Cause of encephalitis from 2002 to 2012: (**A**) the number of patients with and without pathogen detected by year; (**B**) summary of CSF finding by year.

A total of 3099 CSF samples were received from 2002 to 2012, and 337 samples were positive for viruses (10.9%), (Figure 4A). The most frequent diagnoses were CMV with 91 samples (27%) followed by VZV with 79 samples (23.4%), EBV with 72 samples (21.4%), EV with 39 samples (11.6%), HSV-1 with 33 samples (9.8%), HSV-2 with 20 samples (5.9%), and Dengue with 3 samples (0.9%) (Figure 4B). Demographics of individuals with EV encephalitis were obtained from the encephalitis RT-PCR request form, and six (15.4%) were adults (Figure 3).

## 4. Discussion

EV is monitored by DDC under the encephalitis program since 2013. We found that the enterovirus genus is the second most frequent cause of encephalitis from 2013 to 2018, with 17.3%, a marked increase from 11.6% in 2002 to 2012. Interestingly, our data showed an increased prevalence of infection toward adults, with 24.1%, in 2013 to 2018, compared to 13.9% in 2002 to 2012. According to a previous study with the pediatric department, most Thai infants are exposed to enterovirus as early as a few weeks in the community and develop immunity to most enteroviruses before reaching adulthood [14].

The increasing proportion of adult encephalitis caused by EV has also been demonstrated in one of the largest epidemiological studies in the United States [15]. Given its high rate of mutation, an increase in neurotropism could be responsible for the increase in prevalence and trend toward adults [12,13]—firstly, through the hematogenous route with an ability to increase blood–brain barrier permeability through its capsid protein [16], secondly, through the direct route via retrograde axonal transport, and lastly, by trafficking using leukocyte as cargo for access to the CNS [17,18]. Ongoing surveillance of CNS infection caused by EVs will be needed to see if this trend continues.

In this study, we also aimed to improve the diagnosis of encephalitis by using multiplex PCR, which allows wider detection, but results are still not satisfactory. Tropical viruses such as Japanese encephalitis, Rabies, or Nipah are not covered in the PCR panel and probably account for a small fraction of patients [19]. Saraya et al. studied 103 patients with encephalitis in Thailand and found immune causes responsible in as many as 25% [20]. A large fraction of encephalitis unaccounted for in this study are probably from non-infectious etiologies.

There were several limitations in this study. Firstly, diagnosis of encephalitis was based on clinical criteria and at the discretion of the treating physician; thus, other investigations such as lumbar puncture profile and imaging were not available for review. Secondly, the patient’s medical history and medications were unknown, and underlying immunosuppression may have influenced infection with lower virulence organisms. Lastly, there was no information on the disease severity and outcome of these patients.

## 5. Conclusions

There is an increase in the prevalence of encephalitis caused by enterovirus genus with a trend toward adults in Thailand. While the increase in viral neurotropism may be at play, there is currently no direct evidence of increased neurotropism. Nevertheless, clinicians should be aware of the increased prevalence, the possibility of increased pathogenicity, and the usefulness of ongoing active surveillance.
